# Psychometric evaluation of medication safety competence scale for clinical nurses

**DOI:** 10.1186/s12912-021-00679-z

**Published:** 2021-09-09

**Authors:** Zhen Yang, Fengmin Chen, Yingying Lu, Huijun Zhang

**Affiliations:** 1 Department of Nursing, Jinzhou Medical University, No.40, Section 3, Songlu Road, Linghe District, Jinzhou, 121001 China; 2 The First Affiliated Hospital, Jinzhou Medical University, Jinzhou, China

**Keywords:** Medication safety competence, Factor analysis, Medication errors, Psychometric properties, Nurses

## Abstract

**Background:**

Nurses are the most important members of the health care system for medication safety, there are few tools to evaluate their medication safety competence. This study aimed to translate the Medication Safety Competence Scale into Chinese and validate its reliability and validity among clinical nurses.

**Methods:**

A total of 894 clinical nurses were recruited from three cities in China. The original version of the Medication Safety Competence Scale was translated into Chinese using the backward and forward translation procedure. The reliability of the scale was measured by internal consistency, split-half reliability, and stability. The validity of the scale was assessed by the content validity index, exploratory factor analysis and confirmatory factor analysis.

**Results:**

The Cronbach’s coefficient of the scale was 0.940, and the coefficient values for the six domains ranged between 0.843 and 0.948. The split-half reliability and stability were 0.671 and 0.703, respectively. The content validity index of the scale was 0.952. The 6-factor structure, supported by the eigenvalues, total variance explained, and scree plot accounted for 71.485 % of the total variance. Moreover, as a result of the confirmatory factor analysis, the average variance extracted values were 0.55 to 0.70, and the model fitting indexes were all in the acceptable range.

**Conclusions:**

The Chinese version of the Medication Safety Competence Scale had ideal reliability and validity among clinical nurses. The evaluation results of the scale can provide a reference for nursing managers to formulate education plans and intervention measures to improve clinical nurses’ safe medication competence.

## Background

Medication is the largest area in the course of treatment, and it is also the area with the most medical errors. The World Health Organization (WHO) reported that medication errors, as a preventable event, account for 20 % of medical errors and launched global effort to halve medication-related errors in 5 years [1]. Adverse medication safety events may occur in the links of medical advice, nursing medication and patient health education [2, 3]. The investigation showed that drug use errors in medication errors accounted for 50.0 %, followed by drug configuration errors accounted for 18.0 % [4]. Moreover, according to the practice for medication safety issued by the Federal University of Sao Paulo, medication errors or adverse events account for 7.0 % of hospitalizations in the health system, and at least 44,000 to 98,000 deaths per year are caused by medication errors in the United States, which cost 17 billion to 29 billion dollars [5]. Medication safety has become a key topic of global concern.

Medication safety is defined as the protection from accidental injury and the avoidance of any preventable and adverse events during drug use, realising the maximum therapeutic effect and producing the minimum adverse reactions [6]. Meanwhile, it puts forward the operational concept for establishing medication safety through effective assessment, accurate medication selection and use in the right method, dose and time, paying attention to drug contraindications, adverse reactions, interactions, etc., to achieve the purpose of safety, rationality, effectiveness and economy [7, 8]. The purpose of safe medication is to take patients as the center, provide comprehensive medication service for patients, ensure the rationality of medication and improve the health level of patients [9]. Nurses are recognized as the most important group for clinical medication safety globally [10, 11]. In addition, medication safety is an important part of patient safety [12, 13]. Therefore, we should focus on nurses’ positive role in patient safety and medication safety competence under the high prevalence of medication errors.

Medication safety education programs have been developed and implemented among nursing students [14–17]. However, there are few studies on nurses’ medication safety competence in clinical nursing practice, and relevant measurement tools have not been developed in China. Based on the eight stages of scale development and verification [18], Korean scholar Seomun recently combined patient safety with clinical medication nursing in the development of the Medication Safety Competence Scale (MSCS) for nurses in July 2020 [19]. The development of the MSCS has arisen out of this need to assess nurses’ competence on the relevant knowledge, skills, and attitudes for medication safety that can contribute to clinical practice, research, and education. In addition, the scale supported by a six-factor structure provides a comprehensive and effective assessment of medication safety competence from multiple levels and perspectives, which makes up for the deficiency of a single dimensional measurement tool. This study aimed to translate the MSCS into Chinese and validate its reliability and validity among clinical nurses in the context of the high prevalence of medication errors.

## Methods

### Study design and participants

The design of this study was a methodological approach to translate the Medication Safety Competence Scale and to evaluate its psychometric properties with a multicentric cross-sectional survey from March 2021 to May 2021. The sample size was determined using the general rule for factor analytic procedure that requires a minimum of 10 respondents per item [20], but a larger sample is desirable. In this study, 20 respondents per item were required to ensure the accuracy of exploratory factor analysis and confirmatory factor analysis. Participants were recruited by convenience sampling with the assistance of nursing directors from different hospitals. A total of 894 nurses involved in clinical nursing practice were included in the study.

### Instruments

#### General Demographic Characteristics Questionnaire

A thorough literature review was conducted, after which the team designed the General Demographic Characteristics Questionnaire. Participants were required to complete the questionnaire consisting of six items by self-reporting: age, gender, educational level, marital status, site, and professional experience.

#### Medication Safety Competence Scale

Medication safety competence was measured through the Medication Safety Competence Scale developed by Seomun [19]. The scale includes thirty-six items measured on a Likert scale from 1 to 5, corresponding to (1) definitely no, (2) rather no, (3) hard to say, (4) rather yes, and (5) definitely yes. Six domains were evaluated: medication management and patient assessment, improvement of safety problems in the medication process, management of effecting factors, management of safety risks, multidisciplinary collaboration, and responsibility as a professional nurse. The Cronbach’s α coefficient of the scale was 0.96, with those of domains ranging from 0.77 to 0.91.

### Procedures

#### Scale translation procedure

Our translation work has obtained professor Seomun’s permission. First, the Medication Safety Competence Scale was translated into Chinese by two Chinese professors majoring in English language. Then, two Chinese teachers who are native English speakers did the reverse translation. Psychological experts made further adjustments for the translated scale. A preliminary survey using the translated scale was conducted with 12 nurses to verify the clarity and understanding of the scale.

#### Data collection procedure

**Fig. 1:**
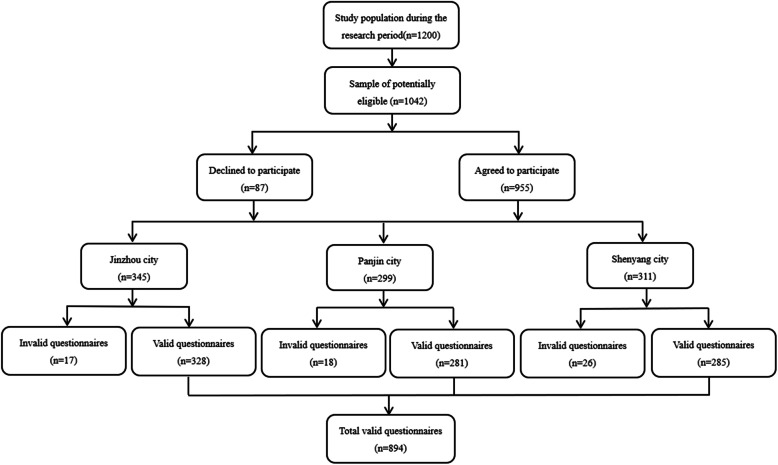
Flowchart of participants

After receiving appropriate training, researchers went to three cities respectively and recruited participates with the assistance of nursing directors. According to the pre-investigation, we were able to contact about 1200 nurses from the three same-level hospitals in the three cities of Northeast China during the study period. 1042 eligible nurses were invited to participate in the cross-sectional survey through convenience sampling, of which 955 agreed to participate. The participants were placed in a quiet classroom to fill out questionnaires anonymously. A total of 894 completed questionnaires were eventually obtained after the removal of invalid ones (Fig. 1).

### Data analysis

Statistical analysis was performed using the SPSS software (v. 22.0) and AMOS (v. 21.0). Continuous data were described as mean (standard deviation), categorical data were expressed as percentages. Mardia’s Skewness & Kurtosis tests were used to determine whether the data have a multivariate normal distribution. When the results of skewness and kurtosis tests are significant (*P* > 0.05) and the standardized multivar -iate kurtosis coefficient is less than 5, the data can be considered to have a multivari -ate normal distribution [21].

#### Items analysis

For items analysis, the total score of the translated scale was ranked from high to poor, and the relationship between the first 27 % (high-score group) and the last 27 % (poor-score group) was analyzed to judge whether the translated scale has an ideal discrimination ability. The correlation between items and the translated scale and the Cronbach’s α coefficient if item deleted were analysed to evaluate whether each item of the translated scale can be retained.

#### Validity analysis

Seven experts were invited to evaluate the content validity of the translated scale using the Delphi method. The content validity index of the items (I-CVI) and the content validity index of the scale (S-CVI) were calculated using the Lawshe’s evaluation method. According to the correlation between each item and the theme, each item was given four ratings of “no correlation” (0 point), “somewhat correlated” (0 point), “quite correlated” (1 point), and “high correlation” (1 point). I-CVI is the ratio of the number of experts who ranked each item with 1 point to the total number of experts. S-CVI is the mean of I-CVI for all items.

To explore and validate the underlying factor structure of the translated scale, an exploratory factor analysis (EFA) and a confirmatory factor analysis (CFA) were performed. The sample of 894 cases was randomly subdivided into two groups, one (n = 447) for EFA and the other (n = 447) for CFA. Kaiser-Meyer-Olkin (KMO) measurement and Bartlett test of sphericity were used to judge the rationality of using principal component analysis (PCA) with varimax rotation in EFA. Varimax rotation is the most commonly used orthogonal technique that minimizes factor complexity with a maximized variance of factor loading. Only when the Bartlett test of sphericity was significant (*P* < 0.05) and the KMO was > 0.60, the dataset was considered appropriate for PCA. The factors were extracted based on the comprehensive consideration of eigenvalues, explained total variance, and the visual inspection of the scree plot. Analysis of Moment Structure (AMOS) was used to confirm the hypothesized factor model in CFA. Convergent validity and discriminant validity were also analyzed for assessing construct validity among item measures.

#### Reliability analysis

The Cronbach’s α coefficients of the translated scale and its dimensions were calculated to assess the internal consistency reliability. The items were divided according to the order of oddness and evenness, the split-half reliability was evaluated by testing the correlation between divided items. Two weeks later, the translated scale was used to assess its stability among 70 nurses. Test-retest correlation analysis was performed to assess the stability and consistency of the scale across the entire period of time during which data were collected. The Intraclass Correlation Coefficient (ICC) was calculated to assess intra- and interrater reliability of the scale.

### Ethical consideration

 All participants were informed of the objectives and the scope of the study and provided their informed consent for participation. All procedures were performed according to the Helsinki declaration of 1964 and its further modifications. The study protocol was approved by the Ethics Committee of the Jinzhou Medical University (LLSC2021213).

## Results

### Descriptive statistics

This study included 894 nurses: 233 males (24.9 %) and 671 females (75.1 %). Participants aged 25 to 34 years accounted for 51.8 %. More than half (61.1 %) of the participants were married; 45.7 % of the participants had an undergraduate education. The proportion of participants who came from Jinzhou city was the largest (36.7 %); for the years of professional experience, 35.0 % of participants have been in clinical care for 6 to10 years. The data have a multivariate normal distribution according to the result of Mardia’s Skewness & Kurtosis tests (*P* > 0.05 and standardized multivaria -te kurtosis coefficient is 3.946). Other sociodemographic informations are shown in Table 1.

**Table 1:** Frequency distribution of demographic characteristics (*n* = 894)

Factors	Group	n	%
Age	18–24	157	17.6
	25–34	463	51.8
	35–44	214	23.9
	≥ 45	60	6.7
Sex	Male	223	24.9
	Female	671	75.1
Education level	Junior college education	257	28.8
	Undergraduate education	409	45.7
	Postgraduate education	228	25.5
Marital status	Unmarried	273	30.5
	Married	546	61.1
	Divorced/Widowed	75	8.4
Site	Jinzhou city	328	36.7
	Panjin city	281	31.4
	Shenyang city	285	31.9
Professional experience (year)	1–5	207	23.2
	6–10	313	35.0
	11–15	181	20.2
	16–20	114	12.8
	≥ 20	79	8.8

### Item analysis

The critical ratio > 3.000 indicated the higher discriminability of items. In this study, the critical ratio of 36 items were 9.818 to 26.010, which indicating that the discrimination ability of each item was good. The scores of each item were positively correlated with the total score (*r* = 0.434 to 0.722, *P* < 0.001), indicated that each item was moderately correlated with the scale. After deleting each item, the Cronbach’s α coefficients value of the translated scale were 0.936 to 0.939, which does not exceed Cronbach’s α value of the scale, indicating that the 36 items could be retained. (Table 2)

**Table 2:** Item analysis for Chinese version of the Medication Safety Competence Scale

Item	Item score (SD)	Critical ratio	Correlation coefficient between item and total score	Cronbach’s Alpha if item deleted
1	2.45 (0.92)	21.968	0.631	0.937
2	2.85 (0.82)	13.757	0.501	0.938
3	2.90 (0.85)	26.010	0.696	0.937
4	2.45 (0.91)	25.444	0.677	0.937
5	2.90 (0.80)	19.573	0.587	0.938
6	2.73 (0.87)	14.737	0.506	0.938
7	2.77 (0.85)	20.833	0.640	0.937
8	2.84 (0.86)	20.145	0.608	0.938
9	2.54 (0.92)	24.320	0.668	0.937
10	2.99 (0.78)	16.657	0.525	0.938
11	3.07 (0.77)	17.242	0.515	0.938
12	3.02 (0.73)	17.523	0.550	0.938
13	3.01 (0.76)	19.855	0.568	0.938
14	3.11 (0.81)	13.113	0.443	0.939
15	3.22 (0.77)	13.587	0.455	0.939
16	3.02 (0.75)	21.111	0.576	0.938
17	2.97 (0.77)	15.407	0.476	0.939
18	3.07 (0.89)	21.692	0.656	0.937
19	3.41 (0.85)	16.659	0.556	0.938
20	3.05 (0.92)	21.643	0.658	0.937
21	2.72 (0.89)	18.939	0.581	0.938
22	3.26 (0.98)	13.309	0.475	0.939
23	3.03 (0.97)	12.677	0.455	0.939
24	2.94 (0.82)	22.123	0.634	0.937
25	2.95 (0.78)	21.565	0.633	0.937
26	2.93 (0.83)	24.758	0.679	0.937
27	2.97 (0.78)	22.670	0.661	0.937
28	3.09 (1.11)	11.511	0.479	0.939
29	2.98 (0.87)	19.314	0.582	0.938
30	2.11 (1.01)	23.893	0.701	0.937
31	2.00 (0.93)	14.884	0.573	0.938
32	2.11 (1.05)	21.692	0.691	0.937
33	2.09 (1.05)	24.522	0.722	0.936
34	3.46 (0.80)	12.767	0.451	0.939
35	3.33 (0.86)	9.818	0.434	0.939
36	3.42 (0.78)	11.415	0.495	0.938

### Validity analysis

#### Content validity analysis

Seven experts independently were invited to evaluate the content validity of the translated scale. The I-CVI and S-CVI of the translated scale were calculated by using the Lawshe’s evaluation method. The results showed that the I-CVIs obtained were 0.857 to 1.000 and the S-CVI was 0.952.

#### Exploratory factor analysis

**Fig. 2:**
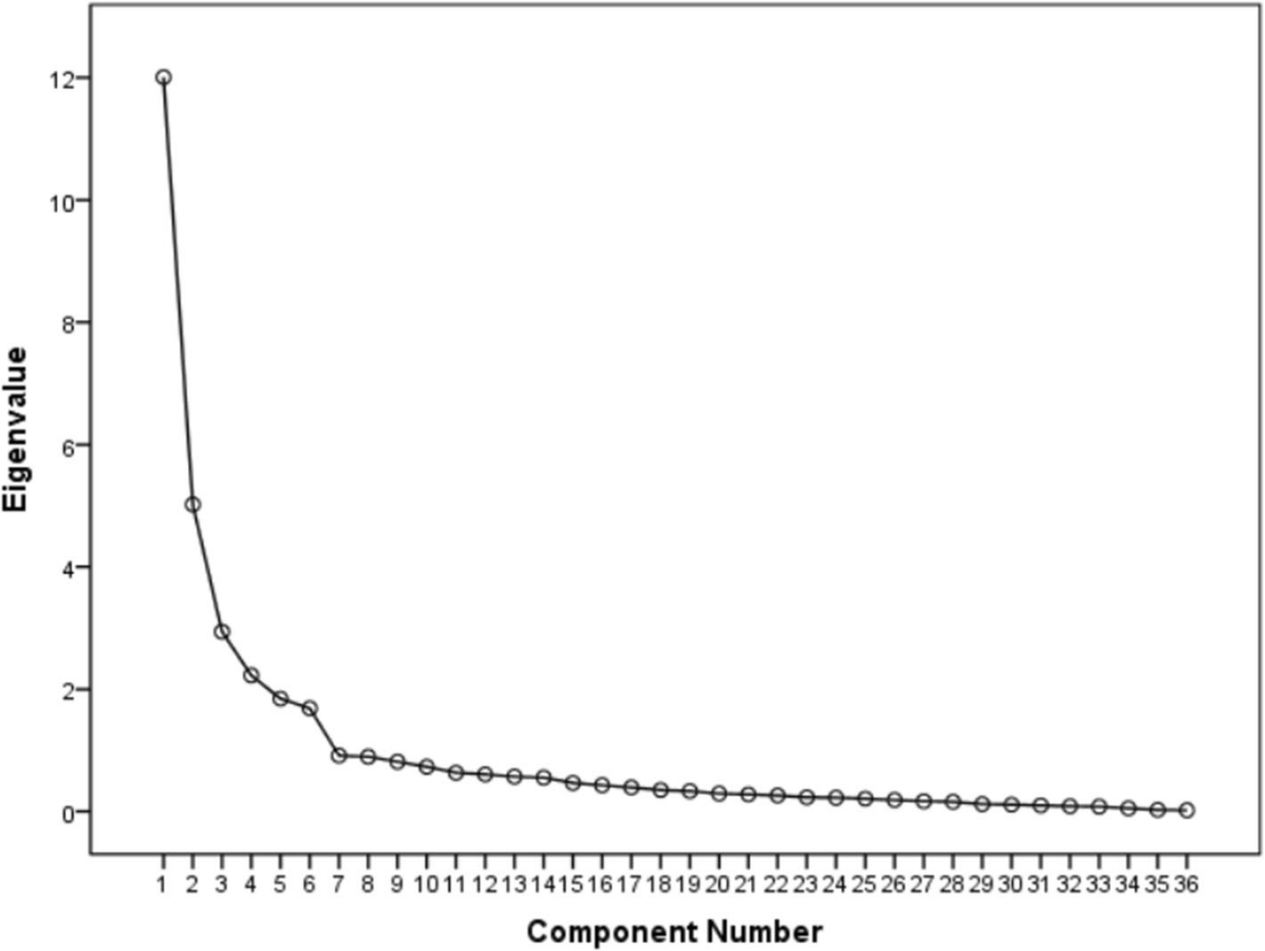
Screen plot of exploratory factor analysis for Chinese version of the medication safety competence scale

The Kaiser-Meyer-Olkin Measure of Sampling Adequacy was 0.853, and the Bartlett test of sphericity was significant (χ^2^ = 32217.515; *P* < 0.001). Therefore, the matrix is not an identity matrix and is appropriate for factor extraction. As a result, 6 factors that explained a total of 71.485 % of the variance had initial eigenvalues > 1 each. The 6-factor structure was further confirmed by the scree plot, as the descending tendency became weak after the sixth point (Fig. 2). After varimax rotation, the six factors explained 6.350 %, 8.495 %, 10.499 %, 12.161 %, 15.928 %, and 18.052 % of the variance. The factor loadings are displayed in Table 3.

**Table 3:** Factor loadings of exploratory factor analysis for the Medication Safety Competence Scale

Item (“I feel confident about. .”)	Factor 1	Factor 2	Factor 3	Factor 4	Factor 5	Factor 6
1. Planning care in the medication process	0.796	-	-	-	-	-
2. Communicating individually according to patients’ condition and level in the medication process	0.814	-	-	-	-	-
3. Evaluating my nursing practice in the medication process	0.757	-	-	-	-	-
4. Giving confidence to patients and caregivers in the medication process	0.828	-	-	-	-	-
5. Giving a sense of stability through clear and consistent communication with patient	0.855	-	-	-	-	-
6. Documentation of assessment, planning, administration of medication, and evaluation of outcomes	0.812	-	-	-	-	-
7. Effective patient training to help patients speak of the symptoms of adverse effects	0.776	-	-	-	-	-
8. Practicing medication care with responsibility for the safety of patients	0.795	-	-	-	-	-
9. Detecting adverse reactions in medication	0.820	-	-	-	-	-
10. Improving the complex and vulnerable way of medication safety (e.g., incorrect administration practices)	-	0.855	-	-	-	-
11. Establish prevention measures when medication errors or near-misses occur	-	0.833	-	-	-	-
12. Trying to create a supportive environment that encourages people to talk about problems when medication errors	-	0.852	-	-	-	-
13. Identifying the root cause rather than blaming the individual when medication errors or near-misses occur	-	0.838	-	-	-	-
14. Establishing prevention measures when adverse drug events occur	-	0.714	-	-	-	-
15. Having a questioning attitude and speaking up when you see something that may be unsafe	-	0.682	-	-	-	-
16. Analyzing the case to find the root cause of the medication error	-	0.888	-	-	-	-
17. Reporting to a nursing manager or supervisor when medication errors or near-misses occur	-	0.756	-	-	-	-
18. Understanding the role of environmental factors such as workflow and resources, which effect medication safety	-	-	-	0.812	-	-
19. Understanding the role of human factors, such as fatigue, that affect medication safety	-	-	-	0.652	-	-
20. Finding information about medication from different sources	-	-	-	0.816	-	-
21. Describing prevention activities for medication safety	-	-	-	0.573	-	-
22. Administration according to the right way (patient, drug, dose, route, and time)	-	-	-	0.792	-	-
23. Using information technology and computerized systems for medication safety	-	-	-	0.758	-	-
24. Coping quickly according to hospital protocol when adverse drug events occur	-	-	0.813	-	-	-
25. Coping quickly according to hospital protocol when medication errors or near-misses occur	-	-	0.825	-	-	-
26. Reporting the adverse drug events according to the reporting system	-	-	0.803	-	-	-
27. Reporting the medication errors or near-misses according to the reporting system	-	-	0.802	-	-	-
28. Assess the need for medication by checking patients’ condition and examination results prior to administration	-	-	0.503	-	-	-
29. Managing the medicine according to the hospital’ s medication management guidelines	-	-	0.809	-	-	-
30. Collaborating with multidisciplinary professionals to address medication safety issues	-	-	-	-	0.793	-
31. Communicating effectively between multidisciplinary members to address medication safety issues	-	-	-	-	0.751	-
32. Sharing decision-making between multidisciplinary to address medication safety issues	-	-	-	-	0.807	-
33. Collaborating with other departments for medication safety	-	-	-	-	0.805	-
34. Receiving regularly medication safety training	-	-	-	-	-	0.790
35. Evaluating regularly my knowledge of medication safety	-	-	-	-	-	0.861
36. Performing medication care with the alertness as the professional	-	-	-	-	-	0.850

#### Confirmatory factor analysis

**Fig. 3:**
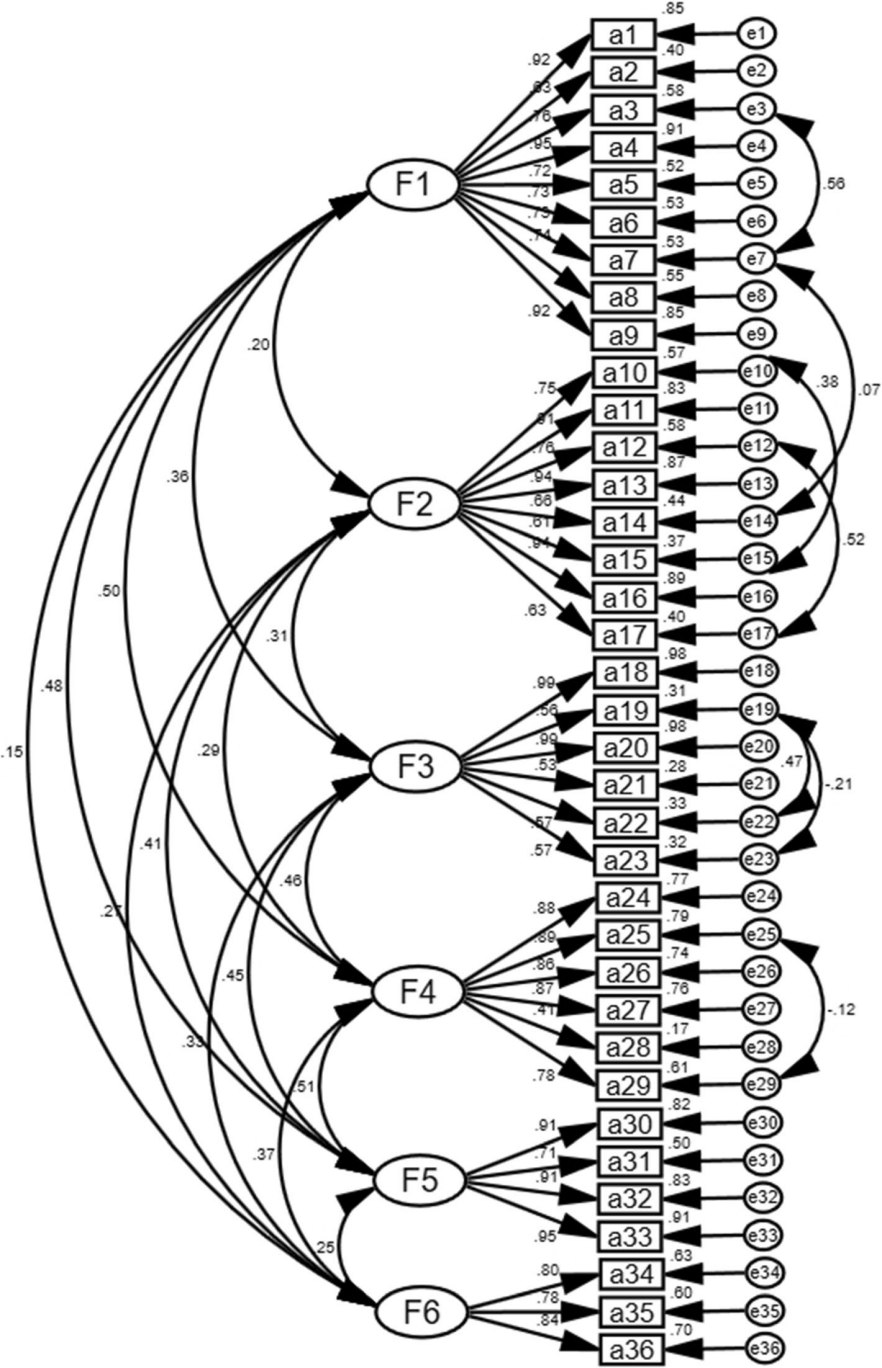
Standardized six-factor structural model of the medication safety competence scale (n=447)

The results of confirmatory factor analysis are shown in Fig. 3. According to the modification indices (MI), the initial model was modified in consecutive steps: e3 and e7, e7 and e14, e10 and e15, e12 and e17, e19 and e22, e19 and e23, e25 and e29, respectively. In the final model fitness index (original model fitness index), the chi-square degree of freedom (χ^2^/df) was 2.939 (5.568), the goodness-of-fit index (GFI) was 0.915 (0.774), the adjusted goodness-of-fit index (AGFI) was 0.901 (0.708), the root mean square error of approximation (RMSEA) was 0.047 (0.084), the tucker lewis index (TLI) was 0.962 (0.795), the comparative fit index (CFI) was 0.969 (0.821), the incremental fit index (IFI) was 0.969 (0.821), the parsimonious goodness-of-fit index (PGFI) was 0.704 (0.612), and the parsimonious normed-of-fit index (PNFI) was 0.775 (0.717). The results of correlation analysis between factors are shown in Table 4. The construct reliability (CR) ranged between 0.87 and 0.94, and the average variance extracted (AVE) values were 0.51–0.77. The correlation between factors ranged from 0.141 to 0.378.

**Table 4:** Results of Confirmatory Factor Analysis

Factor	Item	Parameter significance estimation					Correlation between factors (Discriminant Validity)						Factor Reliability	Construct Reliability	Convergent Validity
		Unstd. Estimate	S.E.	C.R.	P	Std. Estimate	F1	F2	F3	F4	F5	F6	SMC	CR	AVE
F1	a1	1				0.92	1						0.85	0.94	0.63
	a2	0.63	0.03	23.45	< 0.001	0.63							0.40		
	a3	0.76	0.03	24.79	< 0.001	0.76							0.58		
	a4	1.06	0.02	63.98	< 0.001	0.93							0.86		
	a5	0.72	0.03	27.53	< 0.001	0.72							0.52		
	a6	0.74	0.03	29.34	< 0.001	0.73							0.53		
	a7	0.74	0.03	25.82	< 0.001	0.73							0.53		
	a8	0.81	0.03	29.87	< 0.001	0.74							0.55		
	a9	1.07	0.02	45.93	< 0.001	0.92							0.85		
F2	a10	1				0.75	0.180	1					0.56	0.92	0.60
	a11	1.14	0.04	26.89	< 0.001	0.81							0.66		
	a12	0.98	0.03	36.29	< 0.001	0.76							0.58		
	a13	1.18	0.04	27.72	< 0.001	0.94							0.88		
	a14	0.9	0.04	23.77	< 0.001	0.66							0.44		
	a15	0.74	0.03	21.43	< 0.001	0.61							0.37		
	a16	1.28	0.04	29.99	< 0.001	0.94							0.88		
	a17	0.87	0.04	19.69	< 0.001	0.63							0.40		
F3	a18	1				0.95	0.346	0.376	1				0.90	0.85	0.51
	a19	0.55	0.03	20.85	< 0.001	0.56							0.31		
	a20	1.03	0.01	117.89	< 0.001	0.94							0.88		
	a21	0.53	0.03	18.72	< 0.001	0.53							0.28		
	a22	0.63	0.03	20.38	< 0.001	0.57							0.32		
	a23	0.62	0.03	20.41	< 0.001	0.57							0.32		
F4	a24	1				0.88	0.354	0.264	0.316	1			0.77	0.92	0.66
	a25	0.96	0.02	38.41	< 0.001	0.89							0.79		
	a26	0.98	0.03	35.6	< 0.001	0.86							0.74		
	a27	0.93	0.03	36.87	< 0.001	0.87							0.76		
	a28	0.54	0.05	10.76	< 0.001	0.51							0.26		
	a29	0.91	0.03	28.41	< 0.001	0.78							0.61		
F5	a30	1				0.91	0.378	0.307	0.327	0.326	1		0.83	0.93	0.77
	a31	0.72	0.03	26.53	< 0.001	0.71							0.50		
	a32	1.05	0.02	44.62	< 0.001	0.91							0.83		
	a33	1.1	0.02	49.98	< 0.001	0.95							0.90		
F6	a34	1				0.80	0.141	0.254	0.325	0.372	0.227	1	0.64	0.85	0.65
	a35	1.06	0.05	22.81	< 0.001	0.78							0.61		
	a36	1.03	0.04	23.76	< 0.001	0.84							0.71		

Note: *AVE* average variance extracted; *CR* construct reliability; *SMC* Square of Multiple coefficient

### Reliability analysis

The Cronbach’s α coefficient of the translated scale was 0.940, the Cronbach’s α coefficients of the dimensions ranged from 0.843 to 0.948. The split-half reliability was 0.671. After two weeks, 70 nurses were randomly selected for retesting, the test-retest reliability was 0.703 and the ICC was 0.688 (Table 5).

**Table 5:** Reliability analysis for Chinese version of the Medication Safety Competence Scale

The scale and Its dimension	Score (SD)	Cronbach’s Alpha	split-half reliability	test-retest reliability
The Medication Safety Competence Scale	103.73 (17.71)	0.940	0.671	0.703
Patient-centered medication management	24.42 (6.56)	0.948		
Improvement of safety problems	24.41 (5.06)	0.933		
Management of effecting factors	18.53 (4.36)	0.881		
Safety risk management	17.85 (4.19)	0.889		
Multidisciplinary collaboration	8.32 (3.66)	0.927		
Responsibility in the nursing profession	10.20 (2.12)	0.843		

## Discussion

### The Chinese version of the scale has the suitable distinction

The Chinese version of the Medication Safety Competence Scale was obtained in accordance with Chinese guidelines and common expressions. The preliminary survey and the main survey showed that the translated scale was clear and easy to understand. Furthermore, the critical ratio of the items is much better than the standard value [22]. The score of each item was positively correlated with the total score of the scale. The Cronbach’s α coefficient did not exceed the original value of the translated scale after deleting each item, suggesting that all the 36 items should be retained.

### The Chinese version of the scale has suitable validity

Validity refers to the extent to which the measured tool accurately corresponds to the real world [23]. We evaluated the validity of the scale by content and structure analyses. The Delphi method showed that I-CVI and S-CVI were higher than the reference values [24]. It is generally believed that the ideal structure validity can be achieved by two different ways: (1) the factors extracted by exploratory analysis explain 40.00 % or more of the total variation; (2) each item has a high load value of a single factor (> 0.400) and low values of the other factors. In this study, the six factors equivalent to the domains of the scale were extracted by exploratory factor analysis and explained 71.485 % of the total data variation. Moreover, the CFA results confirmed the measurement validity of the translated scale [19]. The construct reliability ranged between 0.87 and 0.94, which was higher than the reference value of 0.70, demonstrating convergent validity [25]. Discriminant validity was also demonstrated because the squares of correlation coefficients between latent variables were smaller than the average variance extracted values [25]. Overall, the Chinese version of the scale showed optimal validity among clinical nurses.

### The Chinese version of the scale has the suitable reliability

Reliability analysis reflects the fact that a scale should consistently reflect the construct it is measuring [26]. We evaluated the reliability of the Chinese version of the scale for three aspects: internal consistency reliability, split-half reliability, and stability. The results showed that the Cronbach’s α coefficient of the translated scale was higher than the original version [19], while the stability of the translated scale was found to be at a very favorable level. Overall, the Chinese version of the scale showed optimal reliability among clinical nurses.

### Limitations

There are some limitations to this study, which should be noted and discussed. First, we did not investigate factors influencing the medication safety competence among clinical nurses, which will be of importance for our future studies. Additionally, self-reporting data contain several potential sources of bias that should be taken into account. Finally, principal component analysis (CFA) was used to extract the common factors in this study, and the results may be overestimated.

## Conclusions

The English version of the Medication Safety Competence Scale has sound psychometric properties and has been successfully introduced into China. The scale will contribute to clinical practice by providing evidence to guide education of nurses in medication safety.

## Implications

Most medication errors are committed by nurses. To solve this problem, it is of great significance for nursing managers to conduct the comprehensive and effective assessment for clinical nurses’ medication safety competence. The developed scale will evaluate the medication safety competence of Chinese clinical nurses, providing an opportunity for development of targeted educational plans.

## Data Availability

The data garnered during the current study and the final dataset used for statistical analysis are available from the corresponding author on reasonable request. The Chinese version of the Medication Safety Competence Scale are available from the corresponding author on reasonable request.
